# Supplementary material for: From sequence to enzyme mechanism using multi-label machine learning
Source: BMC Bioinformatics. 2014 May 19;15:150. doi: 10.1186/1471-2105-15-150 (PMC4229970; doi:10.1186/1471-2105-15-150)
Supplement: Additional file 2 — Java code of ml2db. Additional file ml2db_code.tar.gz contains the Java source code to run the multi-label machine learning experiments and save the results to database. The code’s Javadoc is included. [file 1471-2105-15-150-S2.zip › additional file 2/ml2db/utils/doc/uk/ac/standrews/utils/main/stats/StatUtils.html]

StatUtils


JavaScript is disabled on your browser.


- Overview
- Package
- Class
- Use
- Tree
- Deprecated
- Index
- Help

- Prev Class
- Next Class

- Frames
- No Frames

- All Classes

- Summary:
- Nested |
- Field |
- Constr |
- Method

- Detail:
- Field |
- Constr |
- Method


uk.ac.standrews.utils.main.stats

## Class StatUtils

- java.lang.Object
- - uk.ac.standrews.utils.main.stats.StatUtils

- ---

    

  ```
  public class StatUtils
  extends java.lang.Object
  ```

  Utilities to print simple histograms, percent of frequency etc

  Version:
  :   19 Jul 2008

  Author:
  :   Luna De Ferrari luna.deferrari-at-ed.ac.uk

- - ### Constructor Summary

    Constructors

    Constructor and Description

    `StatUtils()`
  - ### Method Summary

    Methods

    | Modifier and Type | Method and Description |
    | --- | --- |
    | `static java.util.TreeMap<java.lang.Integer,java.lang.Integer>` | `arrayFrequencies(java.lang.Integer[] numbers)` Records the freqencies with which the integers appear in the array |
    | `static java.util.TreeMap<java.lang.Integer,java.lang.Double>` | `fromFrequencyToPercOfFrequency(java.util.TreeMap<java.lang.Integer,java.lang.Integer> frequencies)` Converts a frequency from number of events to percentage over the total number of exctractions |
    | `static int` | `getRandomInt(int upperLimitPlusOne)` Problem: generates a new Random at each call... |
    | `static java.lang.String` | `histogram(java.util.TreeMap<java.lang.Integer,java.lang.Integer> frequencies)` Returns a frequency histogram |
    | `static java.lang.String` | `histogramBar(java.lang.Integer integer, java.lang.Integer frequency)` Returns an histogram bar |
    | `static java.util.TreeMap<java.lang.Integer,java.lang.Integer>` | `paretoFrequencies(Pareto pareto, int numberOfExtractions)` Extracts numbers from a pareto distribution for the given number of times and records the freqencies with which the integers have been extracted |
    | `static java.util.TreeMap<java.lang.Integer,java.lang.Integer>` | `vectorFrequencies(java.util.Vector<java.lang.Integer> vector)` Records the freqencies with which the integers appear in the array |

    - ### Methods inherited from class java.lang.Object

      `equals, getClass, hashCode, notify, notifyAll, toString, wait, wait, wait`

- - ### Constructor Detail


    - #### StatUtils

      ```
      public StatUtils()
      ```
  - ### Method Detail


    - #### arrayFrequencies

      ```
      public static java.util.TreeMap<java.lang.Integer,java.lang.Integer> arrayFrequencies(java.lang.Integer[] numbers)
      ```

      Records the freqencies with which the integers appear in the array

      Returns:
      :   a map containing an integer as key and a frequency as value
          (frequency = number of times the integer has been extracted)


    - #### fromFrequencyToPercOfFrequency

      ```
      public static java.util.TreeMap<java.lang.Integer,java.lang.Double> fromFrequencyToPercOfFrequency(java.util.TreeMap<java.lang.Integer,java.lang.Integer> frequencies)
      ```

      Converts a frequency from number of events to percentage over the total
      number of exctractions

      Parameters:
      :   `frequencies` - the map of each number and the number of times it was
          extracted

      Returns:
      :   the map with each integer as key and the percentage of times it
          was extracted over the total number of extractions


    - #### getRandomInt

      ```
      public static int getRandomInt(int upperLimitPlusOne)
      ```

      Problem: generates a new Random at each call...

      Parameters:
      :   `upperLimitPlusOne` -

      Returns:


    - #### histogram

      ```
      public static java.lang.String histogram(java.util.TreeMap<java.lang.Integer,java.lang.Integer> frequencies)
      ```

      Returns a frequency histogram

      Parameters:
      :   `frequencies` - a map containing an integer as key and a frequency as value
          (frequency = number of times the integer has been extracted)


    - #### histogramBar

      ```
      public static java.lang.String histogramBar(java.lang.Integer integer,
                                  java.lang.Integer frequency)
      ```

      Returns an histogram bar

      Parameters:
      :   `integer` -
      :   `frequency` -

      Returns:
      :   an histogram bar


    - #### paretoFrequencies

      ```
      public static java.util.TreeMap<java.lang.Integer,java.lang.Integer> paretoFrequencies(Pareto pareto,
                                                                             int numberOfExtractions)
      ```

      Extracts numbers from a pareto distribution for the given number of times
      and records the freqencies with which the integers have been extracted

      Parameters:
      :   `numberOfExtractions` - the number of integer extractions from a a pareto distribution

      Returns:
      :   a map containing an integer as key and a frequency as value
          (frequency = number of times the integer has been extracted)


    - #### vectorFrequencies

      ```
      public static java.util.TreeMap<java.lang.Integer,java.lang.Integer> vectorFrequencies(java.util.Vector<java.lang.Integer> vector)
      ```

      Records the freqencies with which the integers appear in the array

      Returns:
      :   a map containing an integer as key and a frequency as value
          (frequency = number of times the integer has been extracted)


- Overview
- Package
- Class
- Use
- Tree
- Deprecated
- Index
- Help

- Prev Class
- Next Class

- Frames
- No Frames

- All Classes

- Summary:
- Nested |
- Field |
- Constr |
- Method

- Detail:
- Field |
- Constr |
- Method
